# Efficacy and safety of T-DM1 in the ‘common-practice’ of HER2+ advanced breast cancer setting: a multicenter study

**DOI:** 10.18632/oncotarget.16373

**Published:** 2017-03-18

**Authors:** Alessandra Fabi, Michelino De Laurentiis, Michele Caruso, Enrichetta Valle, Luca Moscetti, Daniele Santini, Katia Cannita, Luisa Carbognin, Mariangela Ciccarese, Rosalba Rossello, Grazia Arpino, Vita Leonardi, Filippo Montemurro, Nicla La Verde, Daniele Generali, Alberto Zambelli, Giuseppa Scandurra, Michelangelo Russillo, Ida Paris, Anna Maria D'Ottavio, Gianfranco Filippelli, Marianna Giampaglia, Simonetta Stani, Agnese Fabbri, Daniele Alesini, Daniela Cianniello, Diana Giannarelli, Francesco Cognetti

**Affiliations:** ^1^ Oncologia Medica 1, Istituto Nazionale Tumori “Regina Elena”, Roma, Italy; ^2^ Breast Unit, Istituto Pascale, Napoli, Italy; ^3^ Humanitas Centro Catanese di Oncologia, Catania, Italy; ^4^ Oncologia Medica, Ospedale Businco, Cagliari, Italy; ^5^ Oncologia Medica, Ospedale Modena, Modena, Italy; ^6^ Oncologia Medica, Campus Bio-medico Universitario, Roma, Italy; ^7^ Oncologia Medica Ospedale L'Aquila, L'Aquila, Italy; ^8^ Oncologia Medica, Università di Verona, Verona, Italy; ^9^ Oncologia, Ospedale Vito Fazi, Lecce, Italy; ^10^ UOC di Oncologia medica, Ospedale S. Vincenzo, Taormina, Messina, Italy; ^11^ Oncologia Medica, Università Federico II, Napoli, Italy; ^12^ Oncologia medica, ARNAS Civico, Palermo, Italy; ^13^ Fondazione del Piemonte per l'Oncologia, Itituto Tumori Candiolo, Torino, Italy; ^14^ Oncologia Medica, ASST Fatebenefratelli Sacco, PO Fatebenefratelli e Oftalmico, Milano, Italy; ^15^ Dipartimento Universitario Clinico di Scienze Mediche, Chirurgiche e della Salute, Università degli Studi di Trieste, Trieste, Italy; ^16^ Oncologia Medica, Ospedale Papa Giovanni XXIII, Bergamo, Italy; ^17^ Oncologia Medica, Ospedale per le Emergenze Cannizzaro, Catania, Italy; ^18^ Dipartimento Oncologico USL Toscana nord-ovest, Ospedale San Luca, Lucca, Italy; ^19^ Oncologia e Ginecologica Polo Donna, Policlinico A.Gemelli, Roma, Italy; ^20^ Oncologia, Ospedale San Giovanni, Roma, Italy; ^21^ Oncologia P.O. di Paola, ASP di Cosenza, Cosenza, Italy; ^22^ Oncologia Medica, Ospedale San Carlo, Potenza, Italy; ^23^ Oncologia Medica, Ospedale Santo Spirito, Roma, Italy; ^24^ UOC Oncologia, Ospedale Belcolle, Viterbo, Italy; ^25^ Unità di Biostatistica, Istituto Nazionale Tumori “Regina Elena”, Roma, Italy

**Keywords:** HER2, metastatic breast cancer, trastuzumab, taxane, ado-trastuzumab emtansine

## Abstract

Ado-trastuzumab emtansine (T-DM1) is an antibody-drug conjugate approved for the treatment of patients with human epidermal growth factor receptor 2 (HER2)-positive, metastatic breast cancer (mBC). The aim of this ‘field-practice’ study was to investigate the efficacy and safety of T-DM1, focusing on treatment line, previous lapatinib treatment and patterns of metastasis. Three hundred and three patients with HER2-positive mBC who received T-DM1 were identified by reviewing the medical records of 24 Italian Institutions. One hundred fourty-nine (49%) and 264 (87%) had received prior hormonal treatment and/or anti-HER2 targeted therapy, respectively. Particularly, 149 patients had been previously treated with lapatinib. The objective response rate (ORR) was 36.2%, and 44.5% when T-DM1 was administrated as second-line therapy. Considering only patients with liver metastases, the ORR was 44.4%. The median progression-free survival (PFS) was 7.0 months in the overall population, but it reached 9.0 and 12.0 months when TDM-1 was administered as second- and third-line treatment, respectively.

In conclusion, in this ‘real-word’ study evaluating the effects of T-DM1 in patients with HER2-positive mBC who progressed on prior anti-HER2 therapies, we observed a clinically-relevant benefit in those who had received T-DM1 in early metastatic treatment-line and in subjects previously treated with lapatinib.

## INTRODUCTION

Trastuzumab, a humanized IgG1 monoclonal antibody, was the first targeted therapy against the human epidermal growth factor receptor 2 (HER2) showing clinical efficacy in patients with breast cancer [1]. Despite its efficacy, virtually all patients with metastatic breast cancer (mBC) will eventually develop disease progression to trastuzumab, and some early-stage patients will recur after adjuvant treatment, thus making *de novo* and acquired resistance a major clinical issue [2].

Therefore, a number of HER2-directed therapies have been developed with the aim to overcome resistance to trastuzumab, including lapatinib, pertuzumab, and ado-trastuzumab emtansine (T-DM1).

In particular, T-DM1 is an antibody-drug conjugate that combines trastuzumab and DM1 (also called emtansine), a derivative of the anti-microtubule agent maytansin, which is able to deliver highly effective chemotherapy to targeted cells sparing adverse effects [2, 3]. The efficacy of T-DM1 in the second-line treatment of mBC has been shown in the pivotal, phase III open-label EMILIA trial, in which this therapeutic strategy was compared with the combination of lapatinib and capecitabine [4]. With a median follow-up of 19 months, progression-free survival (PFS) was 9.6 months with T-DM1 and 6.4 months with lapatinib and capecitabine, respectively. Moreover, a prolonged overall survival (OS) was also observed in the T-DM1 group (30.9 *vs*. 25.1 months). The most common grade (G) 3/4 adverse events (AEs) in the T-DM1 group were thrombocytopenia (12.9%) and elevated liver transaminases (7.2%), whereas in the control arm were hand foot syndrome (16.4%) and diarrhea (20.7%). Following the results of the EMILIA trial, T-DM1 was the first antibody-drug conjugated approved in cancer treatment, specifically for the therapy of mBC patients who had received trastuzumab and a taxane. In a subsequent phase III study, T-DM1 showed also efficacy as a sequential treatment after lapatinib and trastuzumab (TH3RESA trial) [5, 6].

However, populations included in pivotal trials are often not fully representative of patients encountered in ‘field-practice’; in addition, well-conducted observational studies can complement information provided by clinical trials [7]. To our knowledge, ‘field-practice’ information on T-DM1 is scant [8] and no observational study has specifically investigated the activity of this drug according to a number of factors such as treatment line, prior lapatinib therapy and the location of metastatic sites.

This multicenter ‘field-practice’ study conducted in Italy, investigates the activity and safety of T-DM1, focusing on treatment line, previous lapatinib and pattern of metastasis.

## RESULTS

### Study population

Three hundred and three patients were overall enrolled from April 2012 to June 2016 (Table 1). Median age was 51 years (range 27-78). The majority of patients (*n* = 200; 66%) had an HER2 3+ tumor, as for immunohistochemical (IHC) evaluation. Two hundred and twenty-one (73%) presented visceral metastases. About half of the patients (49%) had received prior hormonal therapies and the majority of patients had undergone an anthracycline-based (96%) or a taxane-based chemotherapy (98%).

**Table 1:** Baseline characteristics (*n* = 303)

Characteristics	Number of patients (%)
Age median (range)	51 (27-78)
**Hormonal Receptors and molecular markers**	
***Estrogen Receptor status***	
Negative	124 (41)
Positive	179 (59)
***Progesterone Receptor status***	
Negative	54 (51)
Positive	149 (49)
**HER2 status**	
IHC* HER2 2+ SISH/FISH^ amplified	103 (34)
IHC* HER2 3+	200 (66)
**Ki67+ cells**	
<20%	52 (17)
>=20%	221 (73)
unknown	30 (10%)
**Previous hormonal therapeutic lines°**	
0	104 (34)
1	88 (29)
2	34 (11.5)
3	18 (6)
>3	9 (3)
unknown	50 (16.5)
**Metastatic Sites**	
Visceral (lung, liver)	221 (73)
Non visceral (bone, soft tissues)	82 (27)
Brain	87 (29)
**Previous chemotherapy metastatic lines**	
0	4 (1.5)
1	69 (23)
2	84 (27)
3	39 (13)
>3	72 (24)
unknown	35 (11.5%)
**Type of previous chemotherapies**	
Anthracycline-based	290 (96%)
Taxane-based	298 (98%)
**Previous Lapatinib lines**	
1	17 (11%)
2	130 (87%)
>3	2 (1%)
**Line of T-DM1 treatment**	
1	8 (2.6%)
2	117 (38.6%)
3	73 (24.1%)
>3	105 (34.7%)

*IHC: immunoistochemistry; SISH/FISH: Silver in situ hybridization/Fluorescent in situ hybridization; °Previous hormonal treatments: tamoxifen, steroidal and no-steroidal aromatase inhibitors

Sixty-nine patients (23%) had received one line of prior anti-HER2 treatment for metastatic disease, 84 (27%) two lines, 39 (13%) three lines and 72 (24%) more than three lines of therapy.

Prior to T-DM1 administration, first- and second-line anti HER2 treatments yielded an objective response rate (ORR) of 60.6% and 46.4%, respectively, and a median PFS of 12 months (range: 2-132) and 8 months (range: 1-82), respectively.

In total, 149 patients were previously treated with lapatinib: of these, 17 (11%) in first-line setting, 130 (87%) as second-line treatment and 2 (1%) in further treatment lines (Table 1).

### Response rate and clinical benefit

A median of 6 cycles (range 1-32) of T-DM1 were administered. Information on response rate was available for 282 patients (Table 2). ORR was 36.2% (102/282), with a total of 11 CRs (3.9%). When T-DM1 was administered in the second-line setting, the ORR increased to 44.5% (52/117), while it dropped to 24.2% (23/95) for treatment beyond third line. With respect to metastatic sites, the highest ORR was reported for liver lesions (44.4%; Supplementary Table S1). The disease control rate (DCR) was 63.8% (180/282). The overall clinical benefit rate (CBR) at 6 months was 53.7% (144/268); CBR was 60.2% in the second-line setting, 54.7% in the third-line setting, and 44.2% beyond the third line.

**Table 2:** Response to T-DM1

Tumor response	I line *n*(%) (*n*=6)	II line *n*(%) (*n*=117)	III line *n*(%) (*n*=64)	>III line *n*(%) (*n*=95)	Total *n*(%) (*n*=282)
Complete Response	0 (0)	6 (5)	4 (6.3)	1 (1,1)	11 (3.9)
Partial Response	1 (16.7)	46 (39)	22 (34.4)	22 (23.1)	91 (32.3)
**Overall Response rate**	**1 (16.7)**	**52 (44.4)**	**26 (40.6)**	**23 (24.2)**	**102 (36.2)**
Stable Disease	5 (83.3)	28 (24)	15 (23.4)	30 (31.6)	78 (27.6)
Progression Disease	0 (0)	37 (32)	23 (35.9)	42 (44.2)	102 (36.2)
Clinical Benefit*	-	60%	55%	44%	53.7%

*Clinical benefit at 6 months

We were interested in studying the response rate to T-DM1 administrated in patients whose last anti-HER2 treatment before T-DM1 was lapatinib (Table 3). A total of 116 patients received T-DM1 at progression of lapatinib. Ten out of the 117 patients (8.5%) who received T-DM1 as second-line treatment had already received lapatinib in the first-line setting; among them, one resistant to lapatinib resulted partially responsive to T-DM1 treatment; of the remaining nine, 3 partially responded to T-DM1 after a stable disease during lapatinib and 6 maintained stable disease as best response. Similarly, among 41 patients who had received lapatinib as second-line therapy and T-DM1 as third-line treatment, 11 (27%) responded to T-DM1, with 2 patients achieving a CR. Among 105 patients who received T-DM1 as fourth-line, 65 received lapatinib in third-line: 15 had progressed on lapatinib, of these, 4 (27%) experienced a response to T-DM1; twenty-five patients showed stability to lapatinib as best response, and 9 of them (36%) responded to T-DM1. The disease control rates of T-DM1 after lapatinib when given as second-line, third-line and fourth-line were 100%, 63% and 77%, respectively.

**Table 3:** Response to lapatinib and T-DM1 treatment (sequential therapy)

Treatment setting	n. of patients	Treatment response (%)			
		PD	SD	PR	CR
I line - lapatinib	10	1 (10)	9 (90)	0	0
II line - T-DM1		0	6(60)	4 (40)	0
	**DCR 100%**				
II line - lapatinib	41	14 (34)	15 (36.5)	12 (29)	2 (5)
III line - T-DM1		13 (32)	17 (41)	9 (22)	2 (5)
		**DCR 63%**			
III line - lapatinib	65	15 (23)	25 (38)	23 (35)	2 (3)
IV line - T-DM1		15 (23)	37 (57)	13 (20)	0
		**DCR 77%**			

CR=complete response; PR=partial response; SD=stable disease; PD=progressive disease

DCR= disease control rate

Thirty-four patients received pertuzumab as first-line therapy and TDM1 as second-line treatment. Three out of 14 patients in progression with pertuzumab achieved stable disease with T-DM1; on the other hand, among 6 patients in stable disease with pertuzumab partially responded to T-DM1.

### Survival

**Figure 1:**
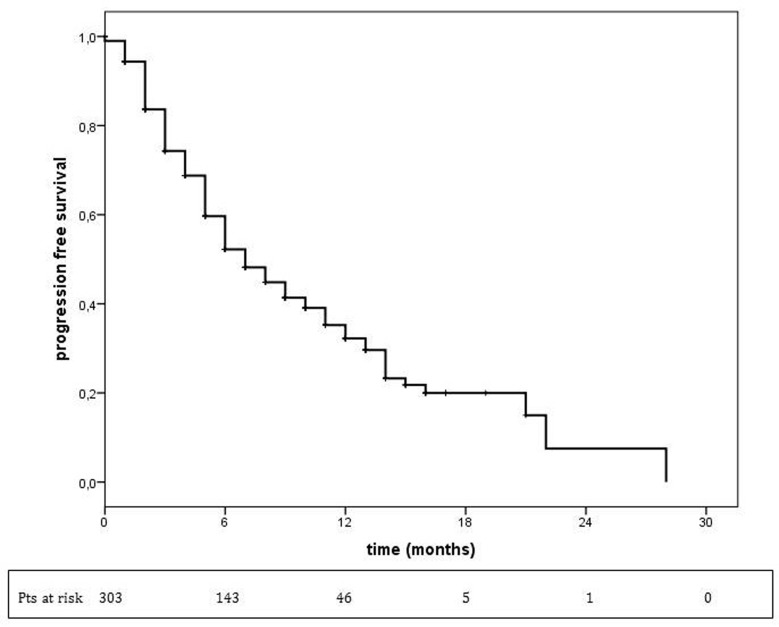
Progression-free survival in overall population treated with T-DM1 T-DM1 treatment median PFS = 7 months (95% CI: 5.8-8.2).

In the overall population, at a median follow up of 10.0 months, (95% CI: 5.8-8.2) (Figure 1).

**Figure 2:**
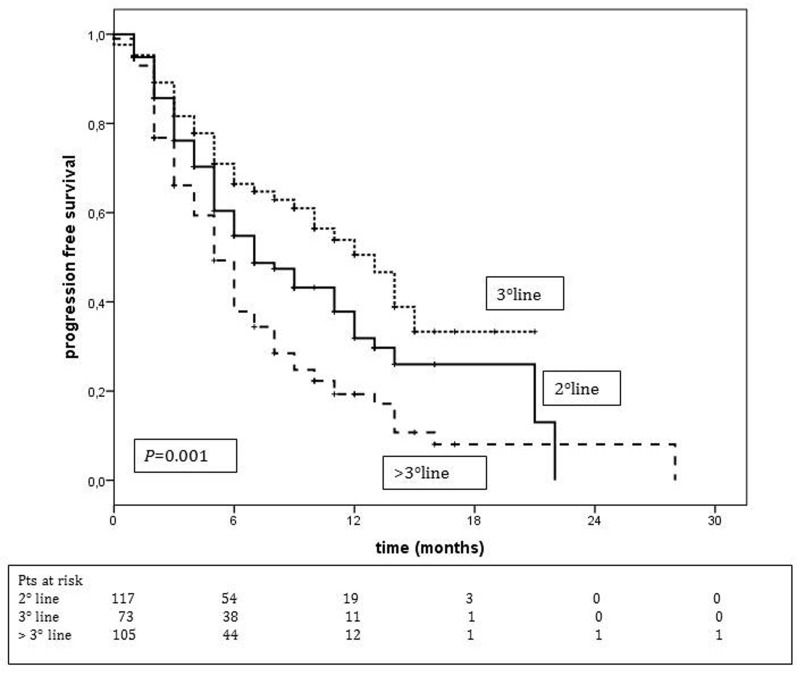
Progression-free survival according to T-DM1 treatment line T-DM1 II line treatment median PFS = 9.0 months (95% CI: 6.4-11.6) T-DM1 III line treatment median PFS = 12.0 months (95% CI: 9.7-16.3) T-DM1 > III line treatment median PFS = 5.0 months (95% CI: 4.0-5.9).

Median PFS was longer when treatment was administered at third line setting (12.0 months, 95% CI: 9.7-16.3, *p* = 0.001) compared with other treatment lines (9.0 months, 95% CI: 6.4-11.6, and 5 months, 95% CI: 4.0-5.9, in the second and beyond the third line of treatment, respectively) (Figure 2).

In the overall population, at a median follow up of 8.0 months, the median overall survival was not reached. The 2-year OS rate was 61%; no difference was seen according to line of T-DM1 therapy (second-line: 61%; third-line: 74%; > third-line: 52%). The 8 patients in which this treatment was administered in the first-line setting were all alive at the time of analysis.

**Figure 3:**
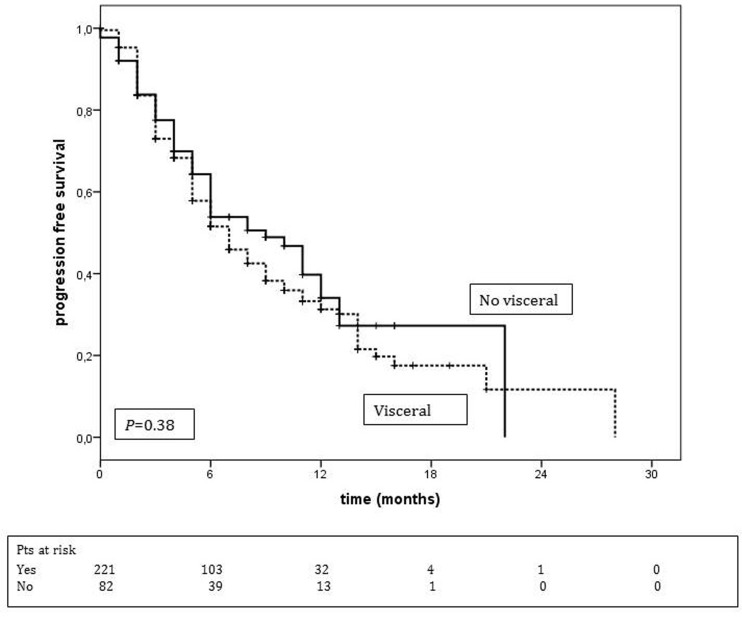
Progression-free survival according to metastatic sites Median PFS of patients with visceral metastases = 9 months (95% CI: 6.2-11.8). Median PFS of patients without visceral metastases = 7.0 months (95% CI: 5.6-8.4).

Comparison of median PFS between patients with and without visceral metastases did not reveal any significant difference, 9.0 months (95% CI: 6.2-11.8) and 7.0 months (95% CI: 5.6-8.4), respectively (*p* = 0.16) (Figure 3). An analysis of PFS among patients with and without brain metastases showed the following: for patients without brain metastases the median PFS was 8 months (95% CI: 5.7-10.3), and the PFS rate was 53% and 37% at 6 and 12 months, respectively. For those with brain metastases the median PFS was 7 months (95% CI: 5.4-8.6) and the PFS rate was 51% and 22% at 6 and 12 months, respectively (*p* = 0.059).

### Safety

All 303 patients were evaluable for safety. Table 4 reports the incidence of adverse events (AEs) in all patients treated with T-DM1. The most common AEs were: asthenia (100/303, 33%), thrombocytopenia (78/303, 25.7%) and increased transaminases (28/303, 9.2%). The majority of AEs were of grade 1 or 2; only 19 grade 3 AEs (alopecia, *n* = 5; thrombocytopenia, *n* = 7; neutropenia, *n* = 2; increased transaminases, *n* = 5) and 3 G4 AEs (thrombocytopenia, neutropenia and transaminase increasing) were reported.

**Table 4:** Adverse events (evaluable in 303 patients)

*N* (%)									
Grade	Neutropenia	Anemia	Thrombo cytopenia	Mucositis	Diarrhea	Transaminases	Neuropathy	Alopecia	Asthenia
1	18 (6)	28 (9)	44 (14.5)	18 (6)	41 (13.5)	18 (6)	17 (6)	4 (1)	64 (21)
2	7 (2)	7 (2)	26 (8.5)	2 (0.7)	6 (2)	7 (2)	4 (1)	8 (3)	33 (11)
3	2 (0.7)	0 (0)	7 (2)	0 (0)	0 (0)	2 (0.7)	0 (0)	5 (2)	3 (1)
4	1 (0.3)	0 (0)	1 (0.3)	0 (0)	0 (0)	1 (0.3)	0 (0)	0 (0)	0 (0)

In total, 41 (13.5%) patients had dose reduction after a median of 5 cycles (range 3-6) mainly for grade 3 and 4 thrombocytopenia, grade 4 neutropenia and increase in transaminases. Only 3 (0.9%) patients required the administration of growth factors for a total of 13 cycles (0.5%).

## DISCUSSION

To our knowledge, this is the first large ‘field-practice’ study to investigate the activity and safety of T-DM1 in an unselected, real-world population of mBC patients previously treated. In particular, we focused our analysis on the effects of T-DM1 according to the line of treatment, a previous lapatinib administration and the presence of visceral metastases.

Although we analyzed an unselected, heavily pre-treated population of mBC patients, our data are fairly consistent with those reported in T-DM1 clinical trials. In the second-line setting, the observed median PFS (9.0 months) and the ORR (44.4%) in our population are similar with the data reported in the pivotal EMILIA study (9.6 months and 43.6%, respectively) [4]. Noteworthy, in our retrospective analysis, the median PFS of patients treated with T-DM1 in third line setting resulted even higher (12 months). In the recently published, phase III MARIANNE study, 1095 patients with previously untreated HER2-positive mBC were randomly assigned to trastuzumab plus taxane (as control), T-DM1 plus placebo, or T-DM1 plus pertuzumab at standard doses [9]. In this study, T-DM1 (alone or in combination with pertuzumab) administrated in the first-line setting, failed to show longer PFS when compared with the control arm. It must be noted that the population enrolled in the MARIANNE trial does not represent the majority of mBC patients encountered in clinical practice. However, a stratification analysis revealed a trend for an increased T-DM1 efficacy in those patients who had received HER2-directed therapy or taxanes in the early breast cancer setting. Consistently with the results of the MARIANNE study, in our study T-DM1 treatment seemed to be associated with the best outcomes when administrated as third line treatment, in heavily pretreated mBC patients. These findings may pave the way for further studies aimed at identifying predictors of response to T-DM1.

Compared with the subgroup of patients exposed to T-DM1 in the second-line setting, most of mBC patients treated with T-DM1 as third line had previously received lapatinib (8.5% *vs*. 56%, respectively). Therefore, we preliminary speculate that the surprisingly longer PFS of T-DM1-treated patients in third-line setting could be associated with prior lapatinib administration. This observation is consistent with the *in vitro* evidence reported by Scaltriti et al [8], suggesting that lapatinib may revert resistance to trastuzumab by yielding the accumulation of HER2 receptors on the surface of breast cancer cell. Although with all the limitations of any retrospective analysis, this finding may suggest, for the first time to our knowledge, more favorable outcomes in patients with prior lapatinib administration and therefore help identifying patients who are more likely to benefit from T-DM1 therapy. Prospective studies on this issue are lacking, and are needed to further investigate this preliminary finding.

Overall, T-DM1 was well tolerated: the incidence rates of AEs of grade 3 or above was low, with only few patients (13.5%) needing dose reduction. This finding is in line with the recent retrospective ‘field-practice’ study by Dzimitrowicz et al [7], which reported a 10% incidence of discontinuation for adverse events. The same study examined the T-DM1 activity as second or further line treatment on mBC patients who had previously received pertuzumab: patients mainly received T-DM1 as fourth-line therapy or subsequent (48%), only 32% of these patients were treated with T-DM1 in the first or second-line setting. Differently, in our population, 34.7% received the drug in fourth line setting or later and 41.2% of patients received T-DM1 treatment in first or second line setting instead. Noteworthy, in the present paper we did not report any subgroup analysis of T-DM1 activity after treatment with pertuzumab/trastuzumab/taxane. However, this analysis will be subject of a dedicated manuscript, which is now ready for submission.

Over the latest years, observational studies are being increasingly used to evaluate the activity of treatments in real-world populations, to fill in data gaps from randomized studies or when randomized studies cannot be conducted [10]. In addition, observational studies can be done with a relatively low cost and often enrolling an unselected population of patients usually not included in clinical studies [10]. However, careful attention must be paid to the observational study design in order to minimize the risk of selection biases [10].

Limitations of our investigation include those shared by all ‘field-practice’ studies such as poor reporting, different clinical approaches between participating Centers and lack of external validation. These limitations could introduce potential bias in the outcome assessment. However, data provided from ‘real world’ data are crucial for a more comprehensive evaluation of the T-DM1 efficacy.

In conclusion, this multicenter, observational study provides ‘real life’ information on the efficacy and safety of T-DM1 in patients with metastatic HER2-positive breast cancer who experienced progression on prior taxane and HER2-neu inhibition approaches. In particular, we observed a potential clinically relevant beneficial effect in patients who received T-DM1 in early lines of treatment and in patients previously treated with lapatinib. To the best of our knowledge this is the first study investigating the activity of T-DM1 treatment in clinical practice on mBC patients who had previously received lapatinib. Additional prospective observational studies are needed to further investigate these findings.

## PATIENTS AND METHODS

The medical records of twenty-four different Italian Institutions were reviewed to identify HER2 positive mBC patients who had been treated with T-DM1. Eligible patients were required to have a diagnosis of HER2 positive tumor. HER2 positivity was determined locally, and defined as 3+ immunohistochemical (IHC) staining (HercepTest; Dako A/S, Glostrup, Denmark) or 2+ IHC staining and HER2/Vep17 Ratio > 2 at fluorescence *in situ* hybridization (FISH) (PathVision HER2 DNA probe kit; Vysis Inc., Downers Grove, IL). Only patients with measurable and/or evaluable disease were included.

No restriction was made on the basis of previous lines of therapies received for metastatic disease. Progressive and/or recurrent disease prior T-DM1 initiation had to be documented in all cases.

Patients who had discontinued HER2-targeted therapies for any cause before the progression of disease were excluded from this analysis.

In Italy, T-DM1 is reimbursed by the Healthcare System; prescriptions are made through a central electronic registry of the Agenzia Italiana del Farmaco (AIFA). This registry mandates tumor restaging every 3 cycles (9 weeks), otherwise not permitting further drug prescriptions. Therefore, all patients in this series had similar restaging timing.

For the purpose of this analysis, an evaluation of the patient's disease based on the Response Evaluation Criteria in Solid Tumors (RECIST) [11] was requested. Overall response rate (ORR) was defined as the proportion of patients achieving complete (CR) or partial remission (PR) among those with measurable disease. Disease control rate (DCR) was defined as the addition of complete, partial responses and stability of disease. Clinical benefit rate (CBR) was considered as the proportion of patients with CR, PR or a stable disease (SD) lasting more than 6 months.

Progression free survival (PFS) was considered as the period elapsing from the initiation of T-DM1 treatment to the date of the first evidence of progressive disease or death due to any cause, whichever occurred first. Survival curves were estimated by the Kaplan-Meier method and differences between curves were evaluated by the log-rank test. For overall survival (OS), patients alive were censored at the date of the last follow-up.

Toxicity was assessed according to the National Cancer Institute Common Terminology Criteria for Adverse Events (version 4.2) grade scaling.

## SUPPLEMENTARY MATERIALS TABLE



## Abbreviations

mBC: metastatic breast cancer
T-DM1: ado-trastuzumab emtansine
HER2: epidermal growth factor receptor 2-positive
IHC: immunohistochemical
FISH: fluorescence *in situ* hybridization
AIFA: Agenzia Italiana del Farmaco
RECIST: Response Evaluation Criteria in Solid Tumors
PFS: progression-free survival
OS: overall survival
AEs: adverse events
G: grade
PD: progressive disease
SD: stable disease
CR: complete remission
PR: partial remission
ORR: objective response rate
DCR: disease control rate
CBR: clinical benefit rate
