# Supplementary material for: Efficacy and safety of T-DM1 in the ‘common-practice’ of HER2+ advanced breast cancer setting: a multicenter study
Source: Oncotarget. 2017 Mar 18;8(38):64481–9. doi: 10.18632/oncotarget.16373 (PMC5610019; doi:10.18632/oncotarget.16373)
Supplement: Supplementary file 1 [file oncotarget-08-64481-s001.pdf]

Efficacy and safety of T-DM1 in the 'common-practice' of HER2+ advanced breast cancer setting: a multicenter study

Supplementary Material

Table S1: Response to T-DM1 according to metastatic site

Activity	N (%)				
	BONE (108)	NODES (96)	LIVER (99)	LUNG (89)	BRAIN* (59)
CR	2 (1.8)	5 (5.2)	6 (6.0)	2 (2.2)	2 (3.3)
PR	25 (23.1)	31 (32.2)	38 (38.3)	26 (29.2)	15 (25.4)
SD	49 (45.3)	33 (34.3)	26 (26.2)	25 (28.0)	11 (18.6)
PD	32 (29.6)	27 (28.1)	29 (29.2)	36 (40.4)	31 (52.5)
ORR	27 (25.0)	36 (37.5)	44 (44.4)	28 (31.5)	17 (28.8)

CR=complete response; PR=partial response; SD=stable disease; PD=progressive disease; ORR=overall response rate

*Evaluable patients for response were 59/87
